# Comparative Genomics Provides Insights into the Genetic Diversity and Evolution of the DPANN Superphylum

**DOI:** 10.1128/mSystems.00602-21

**Published:** 2021-07-13

**Authors:** Liangzhi Li, Zhenghua Liu, Zhicheng Zhou, Min Zhang, Delong Meng, Xueduan Liu, Ye Huang, Xiutong Li, Zhen Jiang, Shuiping Zhong, Lukasz Drewniak, Zhendong Yang, Qian Li, Yongjun Liu, Xiaolong Nan, Biguang Jiang, Chengying Jiang, Huaqun Yin

**Affiliations:** a School of Minerals Processing and Bioengineering, Central South University, Changsha, China; b Key Laboratory of Biometallurgy of Ministry of Education, Central South University, Changsha, China; c Hunan Tobacco Science Institute, Changsha, China; d State Key Laboratory of Microbial Resources, Institute of Microbiology, Chinese Academy of Sciences, Beijing, China; e University of Chinese Academy of Sciences, Beijing, China; f College of Zijin Mining, Fuzhou University, Fuzhou, Fujian, China; g National Key Laboratory of Comprehensive Utilization of Low-Grade Refractory Gold Ores, Shanghang, China; h Department of Environmental Microbiology and Biotechnology, Institute of Microbiology, Faculty of Biology, University of Warsaw, Warsaw, Poland; i College of Agronomy, Hunan Agricultural University, Changsha, China; j 306 Geological Prospecting Party, Hunan Bureau of Geology and Mineral Exploration and Development, Changsha, China; University of Pretoria

**Keywords:** DPANN superphylum, evolution, genome reduction, lateral gene transfer, comparative genomics

## Abstract

DPANN is known as highly diverse, globally widespread, and mostly ectosymbiotic archaeal superphylum. However, this group of archaea was overlooked for a long time, and there were limited in-depth studies reported. In this investigation, 41 metagenome-assembled genomes (MAGs) belonging to the DPANN superphylum were recovered (18 MAGs had average nucleotide identity [ANI] values of <95% and a percentage of conserved proteins [POCP] of >50%, while 14 MAGs showed a POCP of <50%), which were analyzed comparatively with 515 other published DPANN genomes. Mismatches to known 16S rRNA gene primers were identified among 16S rRNA genes of DPANN archaea. Numbers of gene families lost (mostly related to energy and amino acid metabolism) were over three times greater than those gained in the evolution of DPANN archaea. Lateral gene transfer (LGT; ∼45.5% was cross-domain) had facilitated niche adaption of the DPANN archaea, ensuring a delicate equilibrium of streamlined genomes with efficient niche-adaptive strategies. For instance, LGT-derived cytochrome *bd* ubiquinol oxidase and arginine deiminase in the genomes of “*Candidatus* Micrarchaeota” could help them better adapt to aerobic acidic mine drainage habitats. In addition, most DPANN archaea acquired enzymes for biosynthesis of extracellular polymeric substances (EPS) and transketolase/transaldolase for the pentose phosphate pathway from *Bacteria*.

**IMPORTANCE** The domain *Archaea* is a key research model for gaining insights into the origin and evolution of life, as well as the relevant biogeochemical processes. The discovery of nanosized DPANN archaea has overthrown many aspects of microbiology. However, the DPANN superphylum still contains a vast genetic novelty and diversity that need to be explored. Comprehensively comparative genomic analysis on the DPANN superphylum was performed in this study, with an attempt to illuminate its metabolic potential, ecological distribution and evolutionary history. Many interphylum differences within the DPANN superphylum were found. For example, *Altiarchaeota* had the biggest genome among DPANN phyla, possessing many pathways missing in other phyla, such as formaldehyde assimilation and the Wood-Ljungdahl pathway. In addition, LGT acted as an important force to provide DPANN archaeal genetic flexibility that permitted the occupation of diverse niches. This study has advanced our understanding of the diversity and genome evolution of archaea.

## INTRODUCTION

Archaea are ubiquitously distributed in nature, being one of the three major domains of life, and multiple lineages of them have adapted to extreme environments (1, 2). Increasing numbers of studies have shown that archaea functioned as essential promoters of global biochemical circulation (3–5). Archaea are also crucial for gaining insights into the arising as well as the adaptive evolution of life (6–8). Archaea were previously considered exclusively free-living organism rather than symbionts. However, this viewpoint was challenged by the discovery of Nanoarchaeum equitans, a genome-reduced, obligately symbiotic archaeon isolated from a hyperthermophilic region. This microorganism was thought to represent a novel archaeal phylum, referred to as “*Candidatus* Nanoarchaeota” (9). A few years later, the range of symbiotic archaea was further extended by the discovery of archaeal Richmond Mine acidophilic nanoorganisms (ARMAN), another genome-reduced, symbiotic archaeon, from acid mine drainage (AMD). ARMAN was later renamed “*Candidatus* Parvarchaeota” and “*Candidatus* Micrarchaeota” (10–12).

Thereafter, the wide use of cultivation-independent single-cell and metagenomic approaches significantly accelerated the discovery of a larger diversity of nanosized archaeal lineages. For example, single-cell genome sequences of two proposed novel phylum-level groups, known as “*Candidatus* Diapherotrites” and “*Candidatus* Aenigmarchaeota,” were recovered from brackish/fresh water and hydrothermal environments (13). It was then suggested that the above-mentioned archaea with reduced genomes constituted a deep-branching superphylum, collectively referred to as DPANN (an acronym for the nanosized archaeal phyla discovered at that time, including *Diapherotrites*, *Parvarchaeota*, *Aenigmarchaeota*, *Nanoarchaeota*, and “*Candidatus* Nanohaloarchaeota”). “*Candidatus* Nanohaloarchaea,” recovered from hypersaline environments, was at first classified in the DPANN group (13, 14). However, further robust phylogenetic evidence proved that the placement of *Nanohaloarchaea* within the DPANN superphylum was a long-branch attraction (LBA) artifact and that *Nanohaloarchaea* should be reclassified as a member of the superclass *Stenosarchaea* (15). Thereafter, *Nanohaloarchaea* was excluded from the DPANN group (NCBI taxonomy database; last accessed 24 July 2020) (16).

Metagenome-assembled genomes (MAGs) of “*Candidatus* Pacearchaeota” and “*Candidatus* Woesearchaeota” (previously named DHVE-5/6) (17, 18) were recovered from aquifer and groundwater environments. They were also proposed to be members of the DPANN superphylum (19). *Pacearchaeota* and *Woesearchaeota* reside in diverse habitats, including groundwater (19), hydrothermal vents (17), lakes (20), and marine sediments (21). *Woesearchaeota* was also found in the permafrost area (22) and even the human body, having a putative unexplored relation to human health (23). Another potential phylum-level clade, *Altiarchaeota*, first positioned under the *Euryarchaeota*, was later inferred to be a sublineage of the DPANN superphylum (24, 25). The representative member of *Altiarchaeota*, “*Candidatus* Altiarchaeum hamiconexum” (previously named SM1 euryarchaeon), was isolated from a sulfuric spring (26, 27). Members of *Altiarchaeota* can form biofilms that mimic the string-of-pearls configuration (28) and possess special surface-attached hook-like grappling appendages (hami) (29). Single-cell amplified genomes (SAGs) of *Altiarchaeota* were later recovered from temperate environments, including spring, lake, and river sediment (30).

The suggested symbiotic archaeon of *Altiarchaeum*, “*Candidatus* Huberarchaea” (or “*Candidatus* Huberarchaeota”), was recently added to the DPANN superphylum; MAGs of this organism were recovered from Crystal Geyser (United States) (31). The above-mentioned DPANN archaea represents a putative superphylum that is extremely diverse. Tiny cell volumes, reduced genomes with functional genes for biosynthesis of cofactors and amino acids being rarely identified, and obvious gaps in core metabolic pathways have been observed (32, 33). These are typical characteristics of microorganisms with a symbiotic lifestyle. However, *Altiarchaeota* showed the unusual ability to sustain autotrophic growth on carbon dioxide and, potentially, carbon monoxide, formate, or acetate using a modified Wood-Ljungdahl pathway (27). In addition, *Diapherotrites* exhibited genomic evidence for anabolic biosynthesis of multiple carbohydrates, amino acids, lipids, and nucleotides as well as several cofactors (13). These findings highlighted high metabolic diversity within the DPANN group.

Environmental genomics has significantly facilitated the identification and characterization of numerous novel archaeal lineages, such as those belonging to the DPANN group (13, 24, 34). However, unquestionably, many aspects of the DPANN group, including the overall genetic diversity and evolutionary history, have not yet been clearly revealed. In addition, there is still a vast novelty and diversity of species within the DPANN superphylum that await exploration. This “dark matter” might possess unexpected metabolic diversity and fascinating cellular physiology, but it was so new to us that such organisms were usually not recognized by regular probing technology based on rRNA gene sequences. To date, no report has explored the pan-genome and comprehensive phylogenomics of the DPANN superphylum. In this study, we extended previous studies on the gene repertoires and evolutionary history of the enigmatic DPANN superphylum by the investigation of about 600 DPANN genomes. The DPANN genomes studied consisted of 41 DPANN MAGs recovered from metagenomic data sets (four from our AMD metagenome data sets [unpublished] and the other 37 from public metagenomes in the GenBank [35] and JGI-IMG [36] databases) using a binning strategy and 515 publicly available DPANN genomes scavenged from the GenBank, ggKbase, and JGI-IMG databases.

## RESULTS

### Genomic features of DPANN phyla.

Forty-one DPANN MAGs were recovered from metagenomic data sets using a binning strategy. Four of them were recovered from our AMD metagenome data sets (unpublished) and the other 37 from the public metagenomes of the GenBank (35) and JGI-IMG (36) databases (see Table S1 in the supplemental material for detailed information). The average nucleotide identity (ANI) (37) of each MAG to public DPANN genomes (*n* = 515) (Table S2) and the percentage of conserved proteins (POCP) (38) of each MAG relative to its phylogenetically closest public reference genome in reconstructed whole-genome phylogeny (39, 40) were calculated (Table S1; also, see Table S3 at https://doi.org/10.6084/m9.figshare.14806080.v1 and Fig. S1 at https://doi.org/10.6084/m9.figshare.14215811.v3). Results showed that nine MAGs (bin-3, bin-11, bin-21, bin-23, bin-24, bin-28, bin-30, bin-42, and bin-104) belonged to species with currently available public genomes (ANI, >95%; POCP, >50%), while 18 MAGs (bin-1, bin-6, bin-8, bin-12, bin-13, bin-22, bin-27, bin-29, bin-32, bin-33, bin-34, bin-35, bin-36, bin-37, bin-38, bin-39, bin-40, and bin-227) had ANI values of <95% and a POCP of >50%, which indicated that they could be regarded as genomes from unrepresented species within a represented family (38). The remaining 14 MAGs (bin-4, bin-5, bin-7, bin-14, bin-15, bin-16, bin-17, bin-18, bin-19, bin-20, bin-25, bin-26, bin-31, and bin-176) had lower POCP (<50%), suggesting that they belonged to unrepresented families.

10.1128/mSystems.00602-21.1 TABLE S1 General features of 41 metagenome-assembled genomes (MAGs) used in this study. Download Table S1, XLSX file, 0.02 MB. Copyright © 2021 Li et al. 2021 Li et al. https://creativecommons.org/licenses/by/4.0/ This content is distributed under the terms of the Creative Commons Attribution 4.0 International license.

10.1128/mSystems.00602-21.2 TABLE S2 Genomic statistical information and accession of 515 public DPANN genomes used in this study. Download Table S2, XLSX file, 0.1 MB. Copyright © 2021 Li et al. 2021 Li et al. https://creativecommons.org/licenses/by/4.0/ This content is distributed under the terms of the Creative Commons Attribution 4.0 International license.

**FIG 1:**
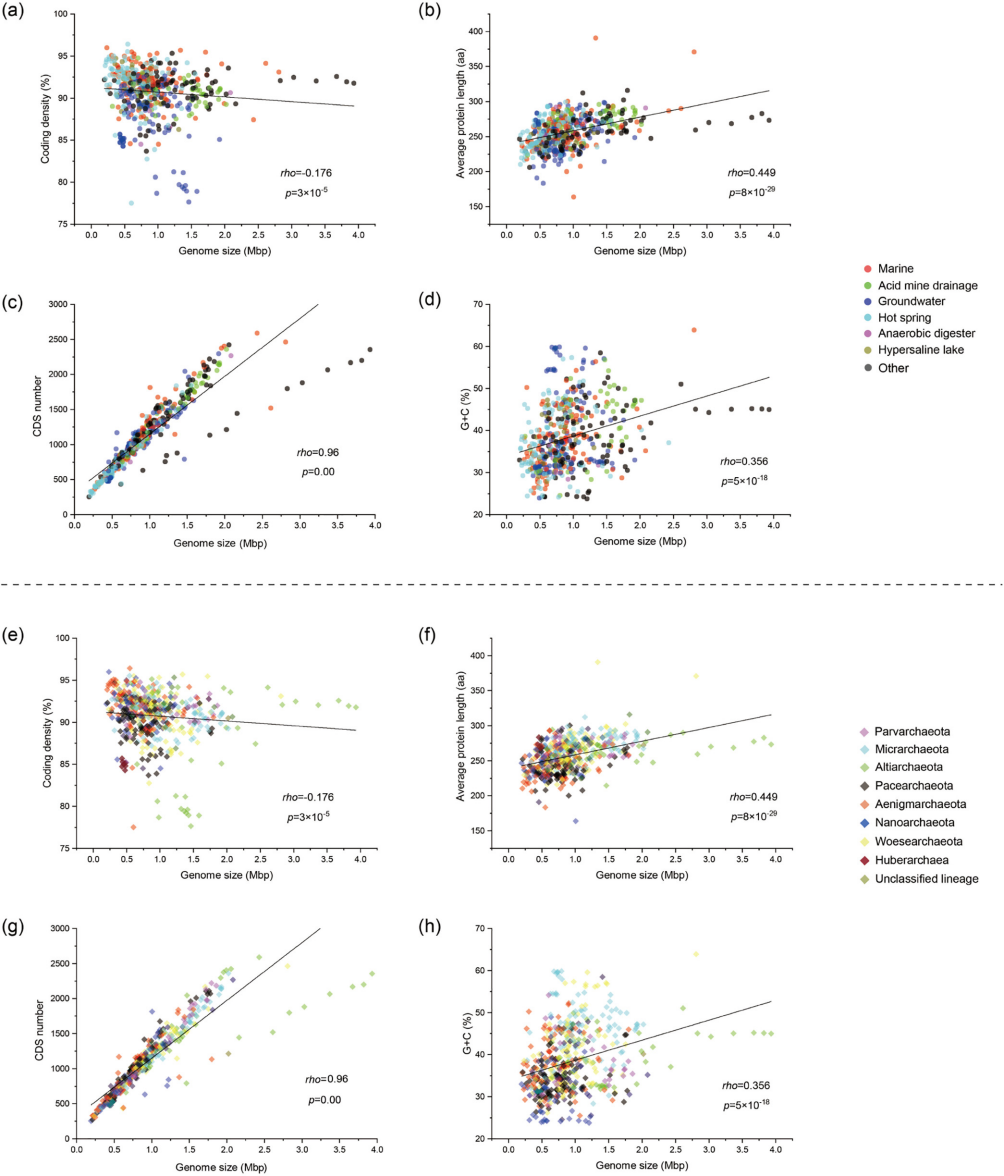
Spearman rank correlation between the genome size and number of CDS, G+C content, and average protein length and coding density, colored according to habitat (top) and taxonomy (bottom) and calculated with OriginPro 2020b. Spearman ρ and the associated *P* value are shown for each scatterplot (a *P* value of ≤0.05 was considered significant).

Among these 41 MAGs, 38 had <5% contamination and 10 had >90% calculated completion and <5% contamination (see Materials and Methods). However, the proposed extensive genome reduction that DPANN archaea underwent might have biased the genome completion assessment (33). These 41 MAGs were analyzed together with 515 publicly available DPANN genomes scavenged from the GenBank, ggKbase, and JGI-IMG databases (Table S2). The genomes of DPANN phyla were generally small in comparison with those of most other archaea (41): *Diapherotrites* (∼1.2 Mbp), *Nanoarchaeota* (∼0.5 Mbp), *Micrarchaeota* (∼1.0 Mbp), *Parvarchaeota* (∼0.8 Mbp), *Altiarchaeota* (∼2.6 Mbp), *Woesearchaeota* (∼1.0 Mbp), *Huberarchaea* (∼0.4 Mbp), *Pacearchaeota* (∼0.7 Mbp), and *Aenigmarchaeota* (∼0.8 Mbp). A significantly positive Spearman correlation (*P* < 0.001) between genome size and G+C content, average protein length, or number of coding sequences (CDS) was observed. Meanwhile, a significantly negative Spearman correlation (*P* < 0.001) between genome size and coding density was found (Fig. 1).

**FIG 2:**
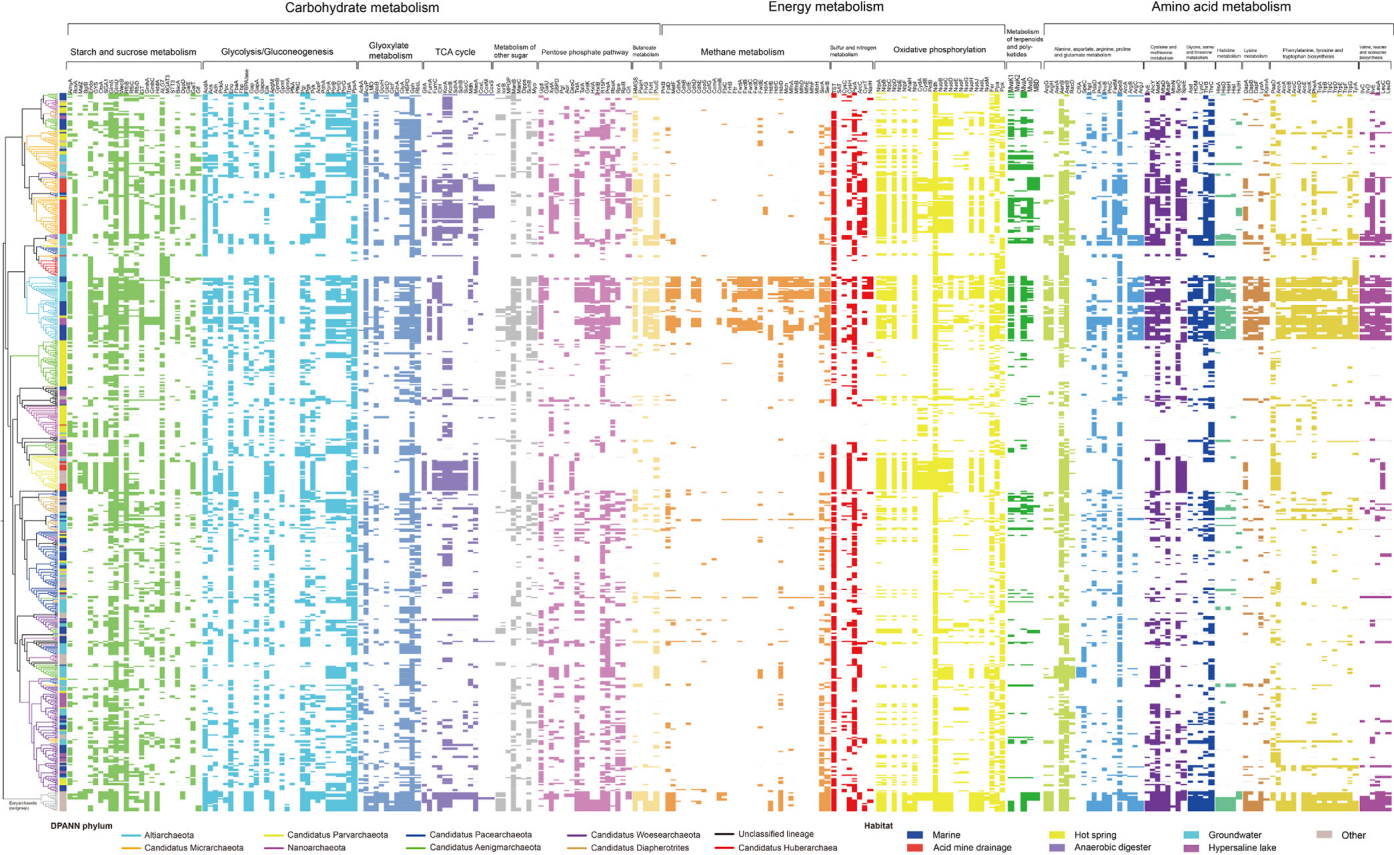
Profile of presence or absence of metabolic or biosynthetic capacities in DPANN archaea and *Euryarchaeota* (outgroup) based on annotation done with eggNOG-mapper v. 2.0 (default parameters: E value < 10^−3^, bit score > 60). The phylogenomic tree shown in the left was constructed based on whole-genome sequences with CVTree3 (k-mer = 4; *Euryarchaeota* was used as outgroup), and the phylogenetic groups were colored according to the original taxonomic assignment in the genome database. The types of habitats from which each genome was recovered are shown in the first bar (on the left) by different colors. The solid and open cells represent the presence and absence of the enzymes, respectively. The cells involved in different pathways are distinguished with different colors. Detailed description for abbreviations is provided in Data S1 at https://doi.org/10.6084/m9.figshare.14806173.v1.

The genomes recovered from a hot spring and a hypersaline lake were significantly smaller than those from other habitats (unpaired *t* test, *P* < 0.05) (see Fig. S2 at https://doi.org/10.6084/m9.figshare.14215802.v2). In addition, genomes of the phyla *Nanoarchaeota* and *Huberarchaea* were smaller than those of other DPANN phyla (unpaired *t* test, *P* < 0.05) (see Fig. S3 at https://doi.org/10.6084/m9.figshare.14215802.v2). Comparative analysis of the pan-genome of each DPANN phylum (the collection of gene families found among genomes of an individual phylum) showed that *Huberarchaea* contained the fewest gene families (729) in its pan-genome. Venn analysis further illustrated interphylum differences in respective pan-genome content (see Fig. S4 at https://doi.org/10.6084/m9.figshare.14215802.v2). Principal-component analysis (PCA) based on the Kyoto Encyclopedia of Genes and Genomes (KEGG) functional categories were performed to reveal the relationship among different members of DPANN archaea (see Fig. S5 at https://doi.org/10.6084/m9.figshare.14215802.v2). From the PCA plot, it was observed that the intergroup difference was significantly greater than the intragroup difference (analysis of similarity [ANOSIM], *R* > 0, *P* < 0.05), and genomes of DPANN archaea appeared to cluster based on their taxonomic assignment rather than their habitat. However, the clustering of *Pacearchaeota*, *Aenigmarchaeota*, and *Nanoarchaeota* was tangled, which was also observed in whole-genome phylogeny (Fig. 2, left). This might result from conserved genes or shared genomic features in these phyla.

Core and pan-genome analyses of the 556 genomes revealed that the pan-genome of the DPANN superphylum reached a size of about 211,966 gene families, fitted into a power-law regression function [*Ps*(*n*) = 423.919*n*^0.983^] with a parameter of γ (0.983) close to 1, suggesting that the pan-genome was still highly “open” (i.e., tended to be linear). The core genome of the DPANN superphylum was fitted into an exponential regression [*Fc*(*n*) = 26457.4e^−3.342^*^n^*], which followed a sharply steep slope and was reduced to 0 gene families within about five genomes. These results indicated vast diversity within the DPANN superphylum and showed that the currently characterized features of this superphylum are still far from saturation.

Omission of DPANN archaea during 16S rRNA-based investigations might be another issue in addressing the diversity of DPANN archaea. Mismatches to 25 known archaeon-specific or universal 16S rRNA gene primers were identified among 16S rRNA genes from available high-quality DPANN genomes (see Fig. S6 at https://doi.org/10.6084/m9.figshare.14215802.v2). Due to mismatches to well-used primers, DPANN sequences are likely neglected during PCR-based investigations. Consistently, we failed to identified *Nanoarchaeota* and *Huberarchaea* sequences in the Sequence Read Archive (SRA) database (with full-length 16S rRNA genes from DPANN genomes as queries), and of the DPANN phyla identified, *Pacearchaeota* had higher abundance (unpaired *t* test, *P* < 0.05) in corresponding samples than other phyla (Fig. S7 [https://doi.org/10.6084/m9.figshare.14215802.v2] and Table S4 [https://doi.org/10.6084/m9.figshare.14806122.v1]).

### Evolutionary analyses of DPANN phyla.

**FIG 3:**
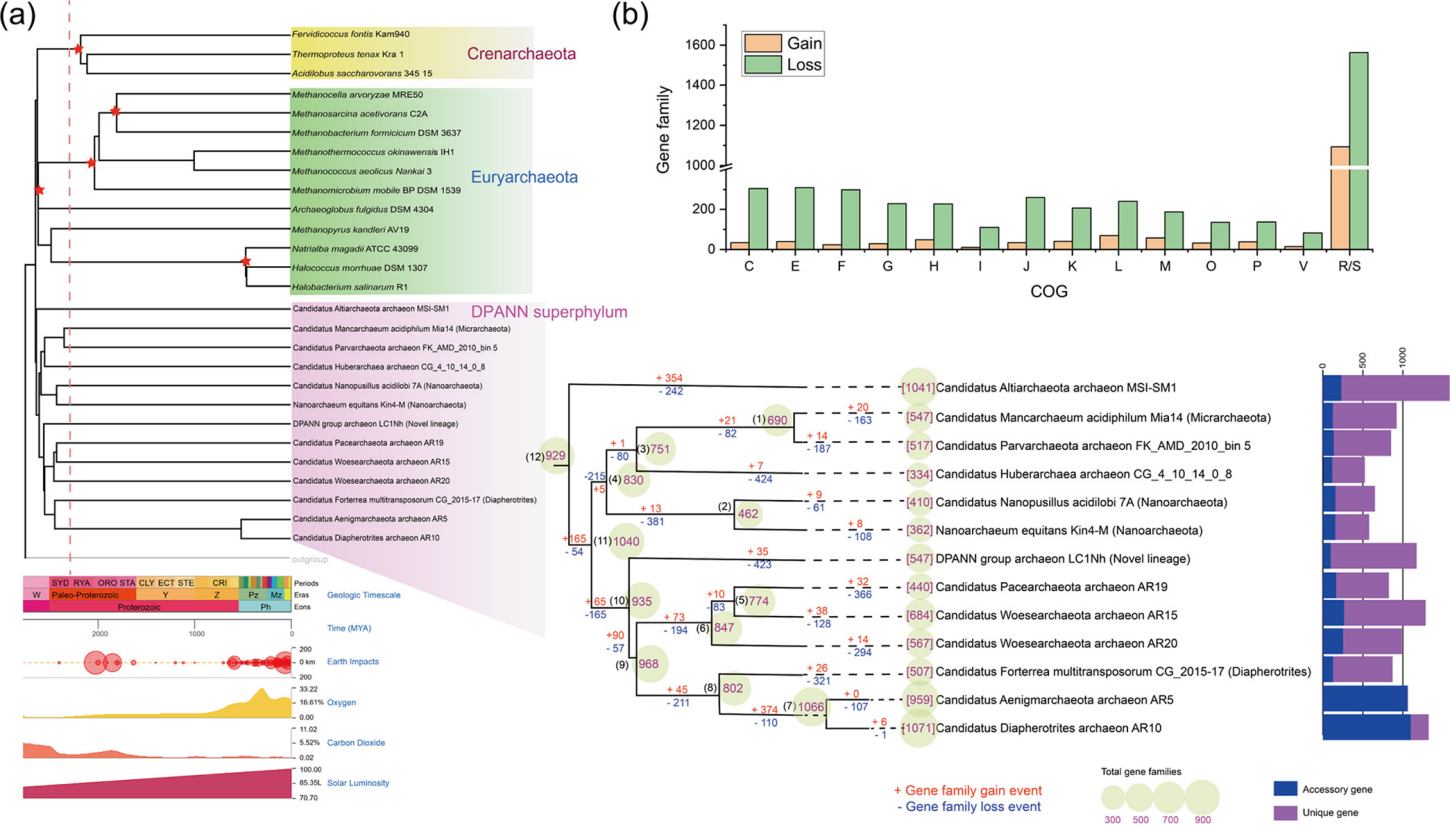
(a) Evolutionary timeline of the DPANN archaea (left) predicted with the RelTime method in MEGA X. Data of asteroid impacts, solar luminosity, and fluctuations of atmospheric oxygen and carbon dioxide amount are displayed synchronously with divergence times in the form of time panels. The estimated occurrence time of the Great Oxidation Event (GOE) (∼2,400 Mya) is marked with a red dotted line. Nodes applying corrections provided by Timetree (http://www.timetree.org) are indicated with a red star. Ancestral genome content reconstruction of DPANN archaea (right) was performed with Dollo parsimony algorithms implemented in the COUNT program. The numbers of gene families of each genome are shown before the names of organisms. The numbers of gene families of the reconstructed respective most recent common ancestor (MRCA) are shown on the nodes. The numbers of gain and loss events are marked on each lineage of the tree. Plus signs indicate gain events, and minus signs indicate loss events. The stacked-bar diagram (right) shows sizes of genes shared by partial genomes (i.e., the accessory genome) and numbers of strain-specific genes (i.e., unique genes). (b) Functional proportions of DPANN gene families undergoing gain and loss events based on COG categories. Detailed description for the COG categories is provided in Data S1 at https://doi.org/10.6084/m9.figshare.14806173.v1.

To gain insight into the evolutionary histories of the DPANN superphylum, gene family gain and loss events were predicted by mapping the identified genes families onto the whole-genome tree (Fig. 3; also, see Materials and Methods). As expected, gene families undergoing loss events dramatically outnumbered those undergoing gain events by a factor of more than 3 (4,457 versus 1,425). The DPANN group was predicted to first diversify around 2,644.56 million years ago (Mya), prior to the occurrence of the Great Oxidation Event (GOE) (∼2,400 Mya) (42) (Fig. 3a). A large number of gene family loss events occurred at branches leading to *Huberarchaea*, *Pacearchaeota* (accounting for 56% and 47% of gene families, respectively), and the most recent common ancestor (MRCA) of *Nanoarchaeota* (accounting for 56% of gene families). The top three clusters of orthologous groups (COG) categories (excluding the poorly characterized proteins) that lost the most gene families annotated were COG category C (energy production and conversion), COG category E (amino acid transport and metabolism), and COG category F (nucleotide transport and metabolism) (Fig. 3b).

### Lateral gene transfer prediction.

**FIG 4:**
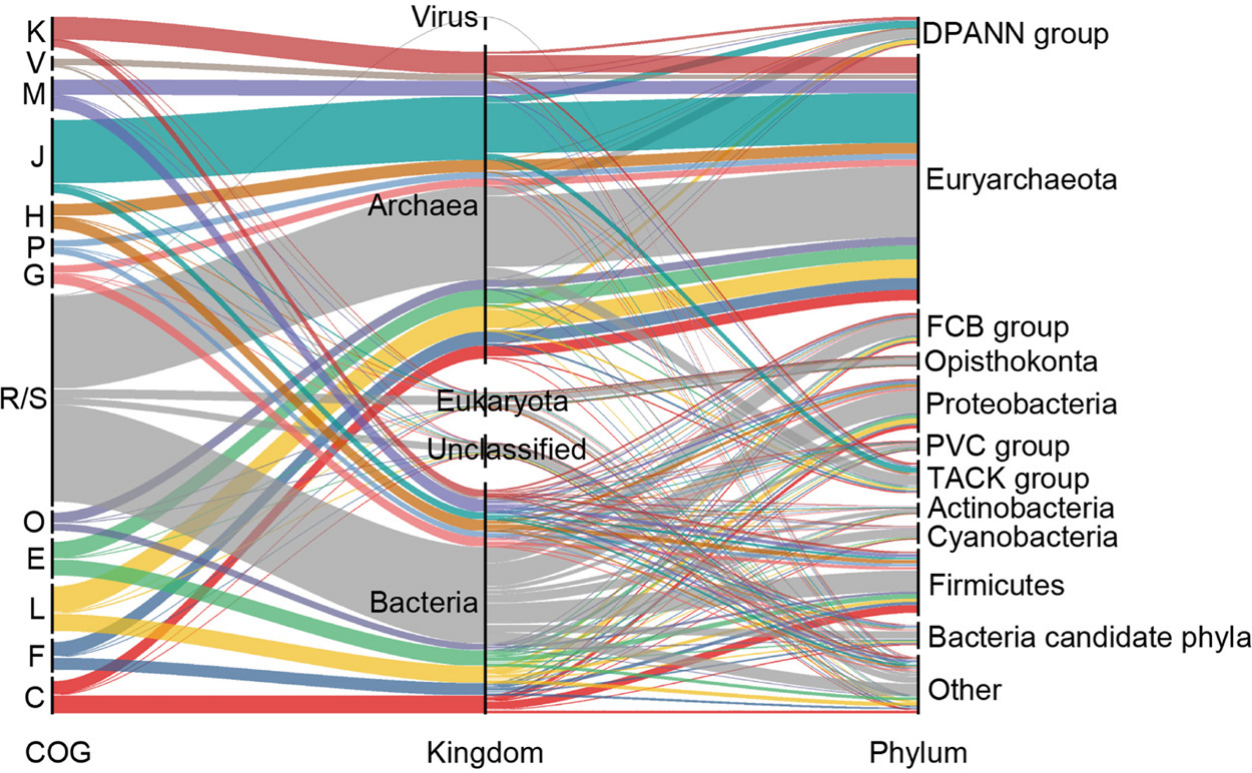
Distributions and relations of COG categories, with the predicted laterally transferred genes (LTGs) annotated and taxonomy of the donors shown. Identification of LTGs was performed through the Integrated Microbial Genomes (IMG) system based on the principles described in Materials and Methods. FCB group = *Fibrobacteres-Chlorobi-Bacteroidetes* superphylum; PVC group = *Planctomycetes-Verrucomicrobia-Chlamydia* superphylum. Descriptions for the COG categories and each LTGs are provided in Data S1 at https://doi.org/10.6084/m9.figshare.14806173.v1 and Table S5 at https://doi.org/10.6084/m9.figshare.14806140.v1.

The predicted laterally transferred genes (LTGs) that the DPANN archaea acquired mostly comprised information processing, defensive, and metabolic functions, with ∼12.3% in COG category J (translation, ribosomal structure and biogenesis), ∼7.4% in COG category L (replication, recombination and repair), and ∼5.5% in COG category E (amino acid transport and metabolism); ∼5.5% of the total lateral gene transfers (LGTs) were in COG category C (energy production and conversion), 5.1% were in COG category K (transcription), and 5.0% were in COG category M (cell wall/membrane/envelope biogenesis) (Fig. 4). Quite a number of the identified potential LGTs (∼45.5%) were cross-domain, of which *Firmicutes* (contributing to 10.6% of LGTs) and *Proteobacteria* (∼9.1%) were major donors. Among the interdomain LGTs, most appeared to be acquired from the *Euryarchaeota* (∼33.6%) and the TACK (*Thaumarchaeota*, “*Candidatus* Aigarchaeota,” *Crenarchaeota*, and “*Candidatus* Korarchaeota”) group (∼5.4%). In addition, LGTs within the DPANN superphylum (∼5.0%) were also detected. These LTGs covered almost all major metabolic pathways, as seen by mapping the function of these LTGs back to KEGG pathways (see Fig. S8 at https://doi.org/10.6084/m9.figshare.14215802.v2).

### Coding potential of DPANN phyla.

**FIG 5:**
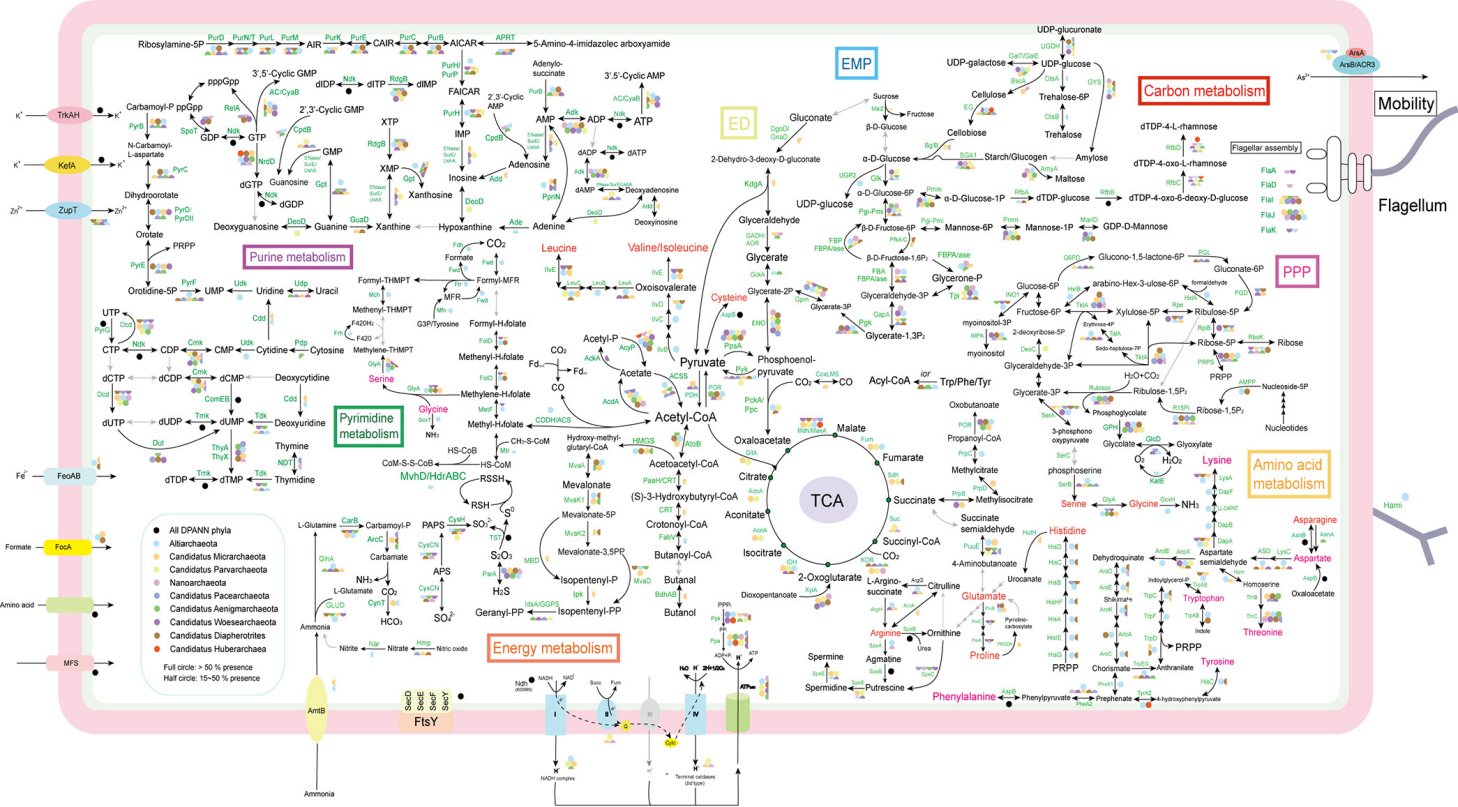
Metabolic reconstruction of major metabolic pathways in DPANN archaea. Annotation was performed with eggNOG-mapper v. 2.0 (default parameters: E value < 10^−3^, bit score > 60). Each phylum of DPANN is depicted as a colored circle. Black arrows indicate that the corresponding proteins were detected for the pathways, whereas gray arrows indicate that the corresponding proteins were not detected. Full circles represent over 50% presence, while half circles represent 15 to 50% presence. Detailed distribution data are provided in Table S6 at https://doi.org/10.6084/m9.figshare.14806212.v1. Detailed description for abbreviations is provided in Data S1 at https://doi.org/10.6084/m9.figshare.14806173.v1.

Metabolic reconstruction of DPANN phyla was also performed (Fig. 2 and 5). Results showed that the metabolic traits of DPANN genomes were a patchwork with few coherent features in most phylum-level radiations, which might represent indirect evidence of the complex evolution of the mysterious DPANN lineages (33). The DPANN archaea were characterized by the absence of biosynthetic pathways for amino acids, lipids, cofactors, vitamins, and nucleotides, but to different extents. In addition, although DPANN microorganisms seemed to have lost genes in many important functional categories, they selectively retained genes related to the central informational processes of DNA replication, transcription, and translation.

### (i) Energy metabolism.

Glycoside hydrolases (GHs; e.g., MalZ, BglB, SGA1, and AmyA) were annotated in ∼37.1% of DPANN genomes, suggesting the ability to utilize complex carbon sources. *Parvarchaeota* possessed more GHs than other phyla (unpaired *t* test, *P < *0.001) (see Fig. S9 at https://doi.org/10.6084/m9.figshare.14215802.v2) but seemed to lack glycolysis enzymes (e.g., GapA, FBA, and Pgk), which rendered its downstream catabolism of glucose unclear. Rhamnose was a major component of the microbial cell wall and extracellular polymeric substances (EPS), which were crucial for cell adhesion and biofilm formation, providing protection against adverse environmental conditions (43). Correspondingly, biosynthesis enzymes for rhamnose (e.g., RfbB, RfbC, and RfbD) were found in ∼76.7% DPANN genomes, which are thought to have been derived via LGT, since they clustered with bacterial homologs in sequence similarity network (SSN) (see Fig. S10 to S12 at https://doi.org/10.6084/m9.figshare.14215802.v2).

6-Phosphofructokinase (Pfk) was widely absent in most DPANN phyla. Only a few genomes of *Woesearchaeota* (*n* = 6), *Diapherotrites* (*n* = 3), *Aenigmarchaeota* (*n* = 8), and *Micrarchaeota* (*n* = 27) possessed the ATP-dependent PfkA or ADP-dependent PfkC. A complete (or nearly complete) pentose phosphate pathway (PPP) was found in ∼42.4% DPANN genomes (e.g., the phyla *Woesearchaeota*, *Pacearchaeota*, and *Altiarchaeota*). The absence of Pfk in DPANN might be compensated for by PPP, which catalyzes the transformation of fructose-6-phosphate to glyceraldehyde-3-phosphate, bypassing the reaction catalyzed by Pfk. These phyla with PPP also comprised type II/III ribulose-1,5-bisphosphate carboxylases (RubisCOs). These RubisCOs were unlikely to participate in the Calvin-Benson-Bassham (CBB) cycle, considering that another essential enzyme of the CBB cycle, phosphoribulokinase, was missing in DPANN genomes. Genes encoding RubisCO colocalized with AMP phosphorylase genes in DPANN genomes (see Fig. S13a at https://doi.org/10.6084/m9.figshare.14215802.v2), suggesting that these RubisCOs functioned in AMP metabolism (44, 45).

SSN analysis showed that the RubisCOs of DPANN (clustering with homologs from *Euryarchaeota*) were in a hub-like position in the SSN that linked to the cluster consisted of unclassified bacterial/archaeal sequences and another cluster containing mostly *Eukarya* sequences (see Fig. S13b at https://doi.org/10.6084/m9.figshare.14215802.v2). The hub cluster in an SSN was suggested to represent a more ancient form from which other clusters derived (46). Additionally, the genomic neighbors of RubisCO and AMP phosphorylase in DPANN archaea were not conserved, suggesting the occurrence of LGT, in line with a previous report (47). Bifunctional fructose 1,6-bisphosphate aldolase/phosphatase (FBPA/ase) represented an ancestral enzyme, contributing to a unidirectional gluconeogenesis pathway that bypassed the formation of the heat-labile intermediate while retaining high activity (48). FBPA/ase was annotated in 52.4% of *Nanoarchaeota*, 52.7% of *Altiarchaeota*, 36.5% of *Aenigmarchaeota*, and 11.2% of *Micrarchaeota*, and SSN analysis showed that the FBPA/ases of DPANN (clustering with *Euryarchaeota*) were also in a hub-like position in the SSN connecting bacteria and *Crenarchaeota* (see Fig. S14 at https://doi.org/10.6084/m9.figshare.14215802.v2).

Aerobic carbon monoxide dehydrogenase (CoxLMS) was detected in 21.4% of *Micrarchaeota* (most of them were from acid mine drainage), which might function in energy production via oxidation of CO gas present in mine areas (49). Archaea generally lacked transketolase and transaldolase for the nonoxidative pentose phosphate pathway (50, 51). However, about 21.6% to 42.4% of DPANN genomes (mostly *Altiarchaeota*, *Diapherotrites*, and *Woesearchaeota*) possessed *Bacteria*-derived transketolase and transaldolase (see Fig. S15 and S16 at https://doi.org/10.6084/m9.figshare.14215802.v2). Key enzymes of the ribulose monophosphate pathway, 3-hexulose-6-phosphate synthase (HxlA) and 6-phospho-3-hexuloisomerase (HxlB) (52), were annotated in 89.1% of *Altiarchaeota* but absent in other phyla. With regard to enzymes catalyzing the decarboxylation of pyruvate to acetyl coenzyme A (acetyl-CoA), the pyruvate dehydrogenase (PDH) complex usually found in aerobes (53) was present in 52.0% of *Micrarchaeota*, while pyruvate:ferredoxin oxidoreductase (POR) was found in 49.4% of *Aenigmarchaeota*, 81.8% of *Altiarchaeota*, and 69.0% of *Diapherotrites*. A nearly complete tricarboxylic acid (TCA) cycle was found in 48.3% of *Micrarchaeota* and 61.7% of *Parvarchaeota*.

Succinyl-CoA synthetase of the TCA cycle was missing in most *Micrarchaeota*. It was postulated that succinate in the *Micrarchaeota* was generated from methylisocitrate via the methylisocitrate lyase (34). Notably, ∼61.8% of *Altiarchaeota* also harbored some TCA enzymes (e.g., IDH, FumA, and Mdh), which might be involved in the metabolism of biochemical intermediates. *Bacteria*-derived FumA (fumarate hydratase class I) and FumC (fumarate hydratase class II) were also found (see Fig. S17 and S18 at https://doi.org/10.6084/m9.figshare.14215802.v2). *Micrarchaeota* and *Parvarchaeota* seemed to have lost FumA while retaining FumC. FumA was reported to be reactive oxygen species (ROS) sensitive, and FumC, in contrast, is ROS resistant (54). This characteristic difference might account for the distinct distribution of these two forms of fumarate hydratase (Fig. 2), since *Micrarchaeota* and *Parvarchaeota* mostly dwell in aerobic mine area-related habitats, while *Altiarchaeota* mostly inhabit anaerobic groundwater environments. In addition, 53.1% of *Micrarchaeota* and 60.5% of *Parvarchaeota* contained the oxaloacetate-decarboxylating enzyme malate dehydrogenase (MaeA), with putative bacterial origins (see Fig. S19 at https://doi.org/10.6084/m9.figshare.14215802.v2). MaeA catalyzes the oxidative decarboxylation of l-malate to pyruvate and potentially converts malate to oxaloacetate (55). It was noteworthy that many phyla devoid of the majority of TCA cycle enzymes (e.g., *Pacearchaeota*, *Woesearchaeota*, and *Nanoarchaeota*) contained 2-oxoglutarate/2-oxoacid ferredoxin oxidoreductase (KOR) (Fig. 2 and 5). This suggests that the KOR family has a wider application than TCA function, like amino acid degradation, as reported for sulfur-dependent hyperthermophilic archaea (56).

About 52.7% of *Altiarchaeota* possessed a complete archaeal version of the Wood-Ljungdahl (WL) pathway. The assimilation of CO_2_ into acetate in *Altiarchaeota* through the WL pathway has been demonstrated by carbon isotopic and transcriptomic analysis (27), indicating an anaerobic autotrophic lifestyle. The heterodisulfide reductase complex (HdrABC) and the non-F420-reducing hydrogenase iron-sulfur subunit (MvhD) were also annotated in 39.1% of *Altiarchaeota*; these might cooperatively form a cytoplasmic MvhD-HdrABC complex to reduce the disulfide of coenzyme M and coenzyme B (CoMS-SCoB) in the final step in methanogenic pathways (57, 58). These enzymes were also likely acquired via LGT (see Fig. S20 and S21 at https://doi.org/10.6084/m9.figshare.14215802.v2).

The heterodisulfide reductase complex might also be involved in sulfur metabolism by catalyzing the interconversion between protein-bound persulfides (RSS^−^) and thiol protein (RS^−^), together with thiosulfate sulfurtransferase (TST), which was detected in 73.4% of DPANN genomes (59, 60). We found that 26.4% of *Altiarchaeota*, 18.3% of *Woesearchaeota*, 20.9% of *Micrarchaeota*, 13.8% of *Diapherotrites*, and 11.2% of *Aenigmarchaeota* putatively possessed the ability to perform the reversible assimilatory reduction of sulfate to sulfite through LGT-acquired adenylyl sulfate (APS) pathway (see Fig. S22 at https://doi.org/10.6084/m9.figshare.14215802.v2) (61). In addition, LGT-derived polysulfide/thiosulfate reductase (PsrA) (see Fig. S23 at https://doi.org/10.6084/m9.figshare.14215802.v2), which converts thiosulfate into sulfide and sulfite, was annotated in 56.6% of DPANN genomes. It was suggested that anaerobes (e.g., *Altiarchaeota*) shuttled electrons between reduced inorganic sulfur compounds as a strategy for oxidative stress resistance (30).

A putative acid stress resistance strategy was also found. Archaeal membrane (composed of isoprenoid-based lipids) was primarily synthesized via the typical mevalonate pathway (62). This mevalonate pathway was detected in 50.5% of *Altiarchaeota*, 39.7% of *Diapherotrites*, 52.8% of *Micrarchaeota*, and 20.0% of *Aenigmarchaeota*. However, there was a difference between the biosynthetic routes that these phyla took. Namely, 16.3% of *Micrarchaeota* contained a unique LGT-acquired mevalonate 3,5-bisphosphate decarboxylase (MBD) (see Fig. S24 at https://doi.org/10.6084/m9.figshare.14215802.v2) to generate the precursor of isoprenoids. MBD is thought to be more efficient at low pH (62), putatively conferring on *Micrarchaeota* the ability to adapt to acid mine drainage habitats.

Regarding the electron transfer chain, NADH-quinone oxidoreductase (complex I) was detected in most *Micrarchaeota* and *Altiarchaeota*, putatively acquired from *Bacteria* (see Fig. S25 at https://doi.org/10.6084/m9.figshare.14215802.v2). The NADH-binding module (NuoEFG), which was previously supposed to be absent in DPANN (27), was also unexpectedly detected in 23.6% of *Altiarchaeota*, 15.6% of *Woesearchaeota*, and 7.8% of *Aenigmarchaeota*. In comparison, genomes devoid of the NADH-binding module might make use of electron donors other than NADH, such as ferredoxin produced by KOR or POR (63). Succinate dehydrogenase/fumarate reductase (complex II) was found in 42.9% of *Micrarchaeota* and 60.5% of *Parvarchaeota*.

LGT-derived cytochrome *bc*_1_ (complex III) subunit PetB (see Fig. S26 at https://doi.org/10.6084/m9.figshare.14215802.v2) was also detected in 60.5% of *Parvarchaeota* and 33.7% of *Micrarchaeota*. Cytochrome *bd* ubiquinol oxidase (CydAB), which has a high affinity for oxygen (64), was detected in only 57.9% of *Parvarchaeota*, 21.4% of *Micrarchaeota*, and 23.8% of *Nanoarchaeota*, with putatively bacterial origins (see Fig. S27 at https://doi.org/10.6084/m9.figshare.14215802.v2). CydAB could generate proton motive force and potentially detoxify ROS (64, 65), which might confer oxygen-utilizing ability to these taxa.

Inorganic pyrophosphatase, polyphosphate kinase, and a five-subunit V/A-type H^+^/Na^+^-transporting ATPase (more streamlined than the nine-subunit ATPase prototype from euryarchaea [66]) were annotated in 82.7% of *Micrarchaeota*, 80.0% of *Altiarchaeota*, 71.1% of *Parvarchaeota*, and 37.9% of *Diapherotrites*. In addition, an LGT-derived F-type H^+^-transporting ATPase was annotated in an *Altiarchaeota* genome (see Table S5 at https://doi.org/10.6084/m9.figshare.14806140.v1). However, we still failed to detect components of the electron transfer chain in ∼60.6% DPANN genomes. They seemed to rely on substrate-level phosphorylation as a major mode of energy conservation, in a symbiosis lifestyle. Correspondingly, various enzymes for the metabolism of fermentation products (e.g., butyrate, lactate, formate, ethanol, and acetate) were annotated. The *fer* gene, encoding ferredoxin (an important electron transfer protein), was detected in most DPANN phyla inhabiting anoxic biotopes (e.g., 74.5% of *Altiarchaeota* and 72.4% of *Diapherotrites*) but seemed to be consistently missing in those from oxic biotopes (e.g., *Micrarchaeota* and *Parvarchaeota* that inhabited AMD-related habitats). The distinct distribution of DPANN ferredoxin genes in different environments might further impact the ferredoxin-dependent pathways, eventually driving the selective distribution of DPANN members, as observed previously in *Woesearchaeota* (67).

### (ii) Amino acid metabolism.

Enzymes that catalyzed the conversion of metabolic intermediate to alanine were rarely detected. Regarding pathways for arginine synthesis, LGT-derived argininosuccinate synthase (ArgG) and argininosuccinate lyase (ArgH) (see Fig. S28 and S29 at https://doi.org/10.6084/m9.figshare.14215802.v2) were annotated in 77.3% of *Altiarchaeota* and 13.3% of *Micrarchaeota* but were absent in other DPANN. Agmatinase (SpeB) was found in 75.8% of DPANN genomes, and ornithine decarboxylase (SpeC) was detected in 31.7% of *Woesearchaeota*, 20.0% of *Aenigmarchaeota*, 19.0% of *Nanoarchaeota*, and 16.9% of *Pacearchaeota*. Arginine deiminase (ArcA, the key enzyme of the arginine deiminase pathway), which produces ammonia as a by-product of acid stress resistance (68, 69), was found in 28.6% of *Micrarchaeota*. In addition, ∼56.6% of *Micrarchaeota* and ∼63.2% of *Parvarchaeota* possessed the ability to produce spermidine and spermine from agmatine. Polyamines such as spermine and spermidine could regulate cellular antioxidant activity and remove ROS (70), which might confer adaptive benefits for *Micrarchaeota* and *Parvarchaeota*, which mostly inhabited acidic niches rich in heavy metals (34). As expected, these adaptive enzymes (i.e., SpeB, SpeC, SpeD, and ArcA) in DPANN archaea were also found to have been acquired via LGT (see Fig. S30 to S33 at https://doi.org/10.6084/m9.figshare.14215802.v2).

A complete pathway for synthesis of lysine or homoserine from aspartate was found in only ∼72.0% of *Altiarchaeota*. A nearly complete pathway for synthesis of histidine from 5-phosphoribosyl 1-pyrophosphate (PRPP) was found in ∼84.5% of *Altiarchaeota*, ∼17.2% of *Diapherotrites*, and ∼9.2% of *Micrarchaeota*. In addition, histidine ammonia-lyase (HutH), which converts histidine to urocanate and ammonia (the initial step of histidine catabolism), was found in 12.2% of *Micrarchaeota*. Complete (or nearly complete) biosynthesis pathways for phenylalanine, tyrosine, and tryptophan were annotated in only ∼78.8% of *Altiarchaeota*. The phenylacetate degradation enzyme ring-1,2-phenylacetyl-CoA epoxidase (PaaD) was found in 68.4% of *Parvarchaeota*, 62.2% of *Micrarchaeota*, and 52.4% of *Nanoarchaeota* but in fewer autotrophic *Altiarchaeota* (7.3%). A complete biosynthesis pathway of proline from glutamate (ProABC) was annotated only in 26.7% of *Altiarchaeota*, 24.1% of *Diapherotrites*, and 11.7% of *Woesearchaeota*. In comparison, 54.1% of *Micrarchaeota* possessed proline dehydrogenase (PRODH), which catalyzes the initial step of proline catabolism. Last, the complete branched-chain amino acid (leucine, valine, and isoleucine) synthesis pathway (e.g., IlvB, IlvC, IlvD, IlvE, LeuA, LeuB, and LeuC) was annotated in only ∼81.2% of *Altiarchaeota* and ∼49.3% of *Micrarchaeota*.

### (iii) Cofactors, vitamins, and nucleotide biosynthesis.

Enzymes for biosynthesis of cofactors and vitamins were present in ∼54.1% of *Altiarchaeota*, ∼26.5% of *Diapherotrites* and ∼21.6% of *Micrarchaeota*, which included cobalamin (CobA, CobB, CobC, CobD, CobN, CobP, and CobU, and CobS), heme (HemC, HemE, HemH, and PduO), riboflavin (RibB, RibE, and RibH), thiamine (IscS, RsgA, THI4, ThiC, ThiD, ThiE, ThiI, ThiL, ThiM, and ThiN), ubiquinone (MenA, UbiX, and UbiB), and vitamin B_6_ (PdxS and PdxT), as well as enzymes for biosynthesis of nicotinate, folate, biotin, methanopterin, and coenzyme A. As expected, the autotrophic *Altiarchaeota* had the most biosynthesis enzymes for cofactors and vitamins, but many parts of these pathways were still highly incomplete (Fig. 2). In comparison, most other DPANN microorganisms might have to depend entirely on environmental sources, in addition to several salvage pathways, to meet their needs for cofactors and vitamins.

Pathways required for *de novo* biosynthesis of nucleotide pyrimidine and purine were annotated in ∼58.7% of *Altiarchaeota*, ∼40.2% of *Diapherotrites*, ∼34.7% of *Woesearchaeota*, ∼33.7% of *Micrarchaeota*, ∼29.5% of *Aenigmarchaeota*, and ∼24.5% of *Pacearchaeota*. It was noted that *Micrarchaeota* was previously regarded as devoid of nucleotide (purine and pyrimidine) biosynthesis ability (34, 71). The molecules ppGpp (GDP 3′-diphosphate) and pppGpp (GTP 3′-diphosphate) are alarmones that regulate cellular activity by the stringent response (72). Correspondingly, we found RelA/SpoT that synthesized and/or hydrolyzed these intracellular signaling molecules in 69.0% of *Diapherotrites*, 41.7% of *Woesearchaeota*, 21.3% of *Pacearchaeota*, and 19.0% of *Nanoarchaeota*, which was also predicted to be acquired from *Bacteria* (see Fig. S34 at https://doi.org/10.6084/m9.figshare.14215802.v2).

### (iv) Genetic information processing.

Functions associated with the core biological processes of replication, transcription, and translation as well as protein folding and stability were also highly conserved in DPANN archaea (see Fig. S35 at https://doi.org/10.6084/m9.figshare.14214500.v2). In addition, 93.2% of tested DPANN genomes contained FtsZ-based cell division systems, attesting that they represented cellular life forms instead of residual DNA (73). Regarding chaperone and repairing molecules, heat shock protein Hsp20 and thioredoxin (TrxA) were found in ∼70.2% of DPANN genomes. Chaperonin GroEL and prefoldin PfdB were found in ∼88.4% of DPANN genomes; chaperonin GrpE, and the heat shock protein 70 (Hsp70), and chaperones DnaJ and DnaK were present in 85.5% of *Altiarchaeota*, 65.5% of *Diapherotrites*, 61.2% of *Micrarchaeota*, 65.8% of *Parvarchaeota*, and 74.2% of *Woesearchaeota*. Previous studies revealed relatively high gene expression and protein abundance of these proteins in symbionts with reduced genomes (74–76), corroborating the viewpoint that these proteins played critical roles in the biology of symbionts (including the DPANN microorganisms). The chaperones widely present in reduced microbial genomes might ameliorate the destructive influences of deleterious substitutions accumulating in symbionts (77), as well as ensuring correct protein folding and stability (78).

Regarding replication functions, only the DNA primase (DnaG), DNA helicase (Mcm), archaeal-type DNA polymerase (Pol), and RNase HI/HII were retained in all DPANN phyla. Interestingly, most *Micrarchaeota*, *Altiarchaeota*, and *Diapherotrites* had additional eukaryotic-type primase homolog, PriSL, which is distinct from the bacterial primase in the catalytic mechanism (79). Regarding transcription functions, subunits of the RNA polymerase, including RpoA, RpoB, RpoC, RpoD, RpoE, RpoH, RpoL, and RpoN, and the initiation transcription factors TFB (transcription factor B) and TBP (TATA-binding protein) were conserved in ∼88.8% of tested DPANN genomes (the RpoL subunit was consistently missing in *Huberarchaea*). Regarding translational functions, only 12 of the 20 standard tRNA synthetases, together with 24 of 53 the small-subunit ribosomal proteins, 24 of the 83 large-subunit ribosomal proteins, rRNA assembly protein SDO1, ribosome maturation protein SDO1, translation initiation factors EIF1, EIF2, EIF5A, and EIF5B, and elongation factors EIF1A and EIF1B, were consistently observed in all DPANN phyla. However, we observed an extensive lack of enzymes conferring functions related to DNA repair and recombination in tested DPANN phyla (see Fig. S35 at https://doi.org/10.6084/m9.figshare.14214500.v2), which is typical in symbiotic AT-rich small microbes (80).

### (v) Cell appendage.

Archaellum proteins, including FlaA, FlaD, FlaI, FlaJ, and FlaK, were annotated in genomes of *Nanoarchaeota* and *Woesearchaeota* (see Fig. S35 at https://doi.org/10.6084/m9.figshare.14214500.v2), indicating the capacity for motility. The expression of flagellar genes was confirmed in *Nanoarchaeota* (81). *Altiarchaeota* do not have archaella; instead, they possess cell surface appendages called “hami,” which are specialized nano-grappling hooks. These appendages are thought to be related to the adaptation of *Altiarchaeota* to the stream environment (82). SSN and genome neighbor analysis using the full-length hami protein of *Altiarchaeota* (82) as the query revealed that the hami proteins of *Altiarchaeota* themselves form a sparse and separate cluster having no reliable analogue in other taxa. The cluster closest to hami was the peptidylprolyl isomerase protein family (see Fig. S36a at https://doi.org/10.6084/m9.figshare.14215802.v2), indicating their putative evolutionary connection. In addition, genes neighboring those encoding hami in *Altiarchaeota* genomes were unconserved (see Fig. S36b at https://doi.org/10.6084/m9.figshare.14215802.v2).

## DISCUSSION

Nineteen years have passed since the first discovery of *Nanoarchaeota* (9), and the omics era has witnessed the rapidly increased identification of DPANN members that were previously unknown (25). In this study, we tried to reveal more genomic details of the enigmatic DPANN superphylum by using a metagenome binning strategy and comparative genomic analysis of the 556 DPANN genomes. Such information would be valuable for illuminating the genomic features, functional repertoire, and evolutionary history of the DPANN superphylum, providing the theoretical foundation for further study on isolation, cultivation, and preservation.

The editorial in the journal *Science* “So Much More To Know” raised over 100 most frontier scientific questions awaiting answers (83), and we considered that three of them were closely related to this study and worthy of discussion here.

The first question was, “Why are some genomes really big and others quite small (compact)?” (83). Our results showed that the most predominant feature of most DPANN genomes was their ultrasmall genomes, which were predicted to have lost genes for a variety of metabolic pathways. Moreover, we found that there was a significant positive correlation between the genome size and number of CDS, G+C content, and average protein length, as well as a significant negative correlation between the genome size and coding density (Fig. 1). The tiniest genomes in the DPANN superphylum were limited to symbionts that relied on archaeal hosts. Only *Nanoarchaeota* (84) and *Micrarchaeota* (71, 85) within the DPANN superphylum were experimentally confirmed as ectosymbiotic archaea. *Woesearchaeota* was inferred to interact with *Methanomicrobia* and *Methanobacteria* based on a co-occurrence network (67), while *Huberarchaea* potentially lived in symbiosis with *Altiarchaeota* based on covarying cell abundance profiles and microscopic imaging (27, 86). In contrast, *Diapherotrites* and *Altiarchaeota* were suggested to be free-living organisms (30, 87).

The questions of whether the remaining members of the DPANN superphylum live a parasitic or free-living lifestyle and, if they are symbionts, what their hosts are still waiting to be addressed. In addition, it remains to be confirmed if members of DPANN phyla that contain ectosymbiotic representatives are all symbionts without exception. Furthermore, members of the DPANN superphylum diversified from each other before the rise of the earth’s oxygen level, and these archaea were predicted to have evolved independently for millions of years (Fig. 3), similar to the endosymbiotic bacteria (88). However, the reasons and routes for DPANN phyla (apart from *Altiarchaeota*) undergoing such massive genome reduction are not yet fully resolved. In fact, almost all microorganisms harbor an innate bias toward gene deletion in their evolution (89). Because of deletional bias, microorganisms with tiny genomes have minimized the amount of noncoding DNA in their genomes, resulting in a very high coding density (89).

Similar to bacterial endosymbionts, host-restricted archaea in the DPANN superphylum, such as *Nanoarchaeota* and *Micrarchaeota*, were suggested to acquire (or share) metabolites such as cofactors, amino acids, and even ATP from their hosts and/or surroundings (32, 71, 81, 90). This situation would render genes of a variety of functional categories in their genomes superfluous, resulting in relaxed selection for these redundant genes (91, 92). Harmful mutations would then accumulate in these genes, followed by pseudogenization and deletion via genetic drift, and the absence of DNA repair and recombination mechanisms would exacerbate these processes (93–95). Several genes might also be actively removed through selection (96). The net result would be the deletion of nonessential genes and probably obligate reliance on their hosts. Functional complementation between these symbiotic DPANN archaea and their confirmed hosts was observed at the genome level (see Table S7a and S7b at https://doi.org/10.6084/m9.figshare.14806215.v1). However, it remains to be determined if these genomic evolution theories of endosymbionts could be applied to archaeal ectosymbionts.

The wide absence of DNA repair machinery might induce more A or T mutations in the genome, as DNA damage (e.g., cytosine deamination and guanine oxidation) usually results in G/C-to-A/T changes (94, 97). This was the probable cause of the increased A and T content in the reduced DPANN genomes (Fig. 1d).

Environmental stresses might also play a part in shaping the reduced genomes of the DPANN archaea. For example, the DPANN archaea inhabiting hot spring environments (with thermophilic lifestyle) had significantly smaller genomes than those from other habitats in this study (see Fig. S2 at https://doi.org/10.6084/m9.figshare.14215802.v2) (unpaired *t* test, *P* < 0.05). It was reported that there was a negative correlation between growth temperature and genome size in thermophilic microbes (98). In other words, tiny genomes were more adaptive at high temperatures, by losing genes with less benefit in order to achieve energetic stress minimization (99–101). In addition to reduced CDS numbers, proteins from DPANN archaea with smaller genome size usually had a shorter average protein length (Fig. 1b) (ρ = 0.449; *P* < 0.001). This is probably due to the adaptive evolution of these proteins through discarding regions that encoded destabilizing substructures (102, 103), which also help cut metabolic cost (104, 105). In addition, results showed that as the genome of DPANN archaea got smaller, the genome became more compact (with increasing coding density) (Fig. 1a) (ρ = −0.176; *P* < 0.001), consistent with previous research on archaeal genome evolution (106).

This is quite different from *Bacteria*. In *Bacteria*, the proportion of the genome consisting of noncoding regions is comparatively constant across a broad range of genome sizes (106, 107). The above-mentioned features were probably associated with adaptive genome streamlining, with members of the DPANN archaea that inhabited hot springs exhibiting the most reduced forms (108, 109). In addition, the DPANN group was predicted to diversify before the Great Oxidation Event (GOE) (∼2,400 Mya) (42), and both oxidized nitrogen and sulfur-based compounds might be at low concentrations prior to the rise of an oxidizing atmosphere. Except for a few phyla (e.g., *Micrarchaeota* and *Parvarchaeota*) that were later exposed to (micro)aerobic environments, most other DPANN phyla were probably consistently confined in anoxic habitats. In this case, most DPANN phyla failed to evolve the TCA cycle and electron transport chain (ETC) components necessary for an aerobic lifestyle, and they were almost unable to perform dissimilatory nitrate reduction and dissimilatory sulfate reduction.

The second open question proposed by the *Science* editorial was, “Why does lateral transfer occur in so many species and how?” (83). Lateral gene transfer (LGT) is an indispensable evolutionary force in prokaryotes that has a massive impact on their genomic diversity and adaptive evolution. Hot springs, hypersaline environments, and acid mine drainage that DPANN archaea inhabit are all potential hot spots for LGT (110–113). Whether LGT events happen gradually and continuously or rapidly in a short period of time is still under debate (111, 114–116). Our results showed that putative LGT events might have contributed substantially to the genome contents of the DPANN superphylum (see Table S5 at https://doi.org/10.6084/m9.figshare.14806140.v1), in line with previous reports (87, 117). The DPANN cells were ectosymbiotic or episymbiotic (symbionts attached to the surface of other cells), distinct from the endosymbionts (symbionts living within other cells). The DPANN cells with an open environment-exposed cellular membrane did not experience isolation (such as those in insect endosymbionts) that separated them from contact with foreign genetic material (81, 118). In fact, there was evidence that the DPANN cells could take up foreign DNA: the genome of *Nanobsidianus* (a *Nanoarchaeota* from a Yellowstone National Park hot spring) was found to harbor genes originating from a virus detected in the same hot spring (84, 119).

In addition, LGT events might have facilitated niche adaption of the DPANN archaea, allowing for the delicate equilibrium of a streamlined genome with efficient niche-adaptive strategies. For example, the acquisition of cytochrome *bd* ubiquinol oxidase and arginine deiminase might confer on *Micrarchaeota* adaptive advantages under acidic (micro)aerobic conditions. Putative LGT events between symbiotic DPANN archaea and their hosts were also found, in line with previous reports (71, 117). The direct cell-cell contact between the DPANN archaea and their hosts might have provided a good opportunity for gene exchange, as seen between *Nanoarchaeota* and their crenarchaeotal hosts (120). Lateral gene transfer from symbionts into host genomes might also have contributed to gene loss in the DPANN archaea (121), similar to that of bacterial endosymbionts (122–124), and there were indeed genes in genomes of DPANN hosts that were putatively transferred from DPANN donors (see Table S7c at https://doi.org/10.6084/m9.figshare.14806215.v1).

The third open scientific question was, “Who was LUCA (the last universal common ancestor)?” (83). Putative ancestral traits of LUCA include living an anaerobic lifestyle with a Wood-Ljungdahl pathway (125, 126), inhabiting thermal environments rich in transition metals and FeS (e.g., hot springs, deep-sea hydrothermal vents, and probably acid mine drainage) (125), and the presence of FBPA/ase (48). Consistent with this, our results showed that members of the DPANN superphylum contain all these features. SSN analysis showed that the FBPA/ases of DPANN (clustering with the *Euryarchaeota*) were in a hub-like position from which homologs of bacteria and *Crenarchaeota* derived (see Fig. S14 at https://doi.org/10.6084/m9.figshare.14215802.v2). This indicated that DPANN archaea occupied a more ancient position in evolution.

Other ancient characters in DPANN archaea were also revealed by previous studies, such as the presence of split genes (127–129) and the possibility that the DPANN superphylum might be at or close to the phylogenetic root of life (130) or archaea (126). The archaeal ancestor was inferred to possess a relatively simple and small genome, which increased in complexity subsequently and gradually through lateral gene transfer (LGT) and gene duplication (41, 126). Correspondingly, these features (i.e., small genomes and occurrences of LGT) were found in DPANN archaea, as discussed above. Taken together, these observations led us to believe that detailed characterization of the DPANN superphylum would provide more clues to help unravel the mystery of LUCA.

Cultivation-independent genomic approaches have brought dramatic improvements to our understanding of the genome characteristics of the DPANN archaea. However, most species and many features of this superphylum remain unexplored, considering the widely “open” pan-genome of the DPANN archaea and mismatches in 16S rRNA genes against widely used primers. The continuing exploration of the dark matter within this supergroup of archaea will be the focus of further studies. In addition, cultivation studies are also important for characterizing the physiology and morphology and examining the coding potential of the DPANN archaea. To date, only a few strains of DPANN phyla have been able to be cultivated. It is foreseeable that genomics-guided isolation of the uncultured DPANN archaea will be performed extensively in the future, leveraging available genomic information to infer suitable cultivation conditions for the isolation, as seen with the *Nanoarchaeota* (81) and “*Candidatus* Lokiarchaeota” (131).

### Concluding remarks.

In this study, we performed a comparative genomic analysis of about 600 DPANN genomes, including 41 DPANN MAGs recovered from metagenomic data sets (18 MAGs had ANI values of <95% and a POCP of >50%, while 14 MAGs showed a POCP of <50%). We found that there were significant differences in gene repertoire among DPANN phyla, and there was a significant positive correlation between the genome size and number of CDS, G+C content, and average protein length, as well as a significant negative correlation between genome size and coding density. Predicted lost gene families outnumbered those gained by a factor of more than three during the evolution of the DPANN superphylum, whereas the top three COG categories that lost the most gene families annotated were COG category C, COG category E, and COG category F.

LGT (∼45.5% was cross-domain) has promoted adaptive evolution of the DPANN archaea, permitting a delicate equilibrium of streamlined genomes with excellent niche-adaptive strategies. We also found blurred taxonomic boundaries in DPANN phyla and mismatches to known 16S rRNA gene primers among 16S rRNA genes of DPANN archaea, suggesting there were yet largely undetected and uncultivated branches. The insights gained in this study would be helpful for uncovering the genomic diversity of the DPANN superphylum and the evolutionary adaptation of these miniature archaea to such a broad range of environmental conditions, providing hints for further study on their detection, isolation, and cultivation.

## MATERIALS AND METHODS

### Sample collection, sequencing, and assembly.

Six metagenomic samples with the identifiers C1W, C3W, C4W, C5W, C6W, and C9W were collected from six individual stations in the acid mine drainage (AMD) of DaBaoShan, Guangdong Province, China (with a latitude and longitude range of 24.554 to 24.557 N and 113.721 to 113.723 E, an altitude range of 598.84 to 641.90 m, a temperature range of 32.8 to 38.2°C, a pH range of 2.38 to 2.59, and a dissolved oxygen range of 4.92 to 6.14 mg/liter). Total environmental genomic DNA was extracted from these AMD samples using the PowerPlant DNA isolation kit (Mo Bio Laboratories, CA, USA) following the manufacturer’s instructions. First, DNA samples were sheared into smaller fragments by nebulization. Then, the overhangs resulting from fragmentation were converted to blunt ends by using T4 DNA polymerase, Klenow fragment, and T4 polynucleotide kinase. After addition of an A (adenine) base to the 3′ end of the blunt phosphorylated DNA fragments, adapters were ligated to the ends of the DNA fragments. Then, short fragments were removed with Ampure beads.

An Agilent 2100 Bioanalyzer and ABI StepOnePlus real-time PCR system were used to qualify and quantify the sample libraries. The qualified libraries were then sequenced on an Illumina HiSeq platform at Shenzhen BGI Gene Co., Ltd. (Shenzhen, China). In order to obtain more accurate and reliable results, unqualified reads were removed to obtain clean data. The unqualified reads were defined as follows: (i) reads containing 10% or more ambiguous bases (N base); (ii) reads containing adapter sequences (default: 15 bases overlapped by reads and adapter); (iii) reads containing 50% or more low-quality (*Q* < 20) bases. Preprocessed reads were assembled with IDBA_UD v.1.1.1 (132) to obtain longer contigs, and reads were assembled with a series of different-size k-mers in parallel. Reads were mapped back to each assembly result with SOAPdenovo2 (133). The optimal k-mer size and assembly results were chosen depending on both contig *N*_50_ and mapping rate. During the assembly process, only contigs of no less than 300 bp were kept for further analysis.

### Metagenome-assembled genome reconstruction.

Binning strategies provided by both Maxbin v.2.0 (134) and MetaBAT v.0.32.4 (135) were applied for metagenome-assembled genome (MAG) recruitment from our six AMD metagenome data sets (unpublished data) as well as 36 publicly available metagenomes obtained from the GenBank and JGI-IMG databases through database mining (see Table S1 for metagenome accession numbers and other information). After that, the 41 bins acquired were refined with Prinseq (136). The phylogenetic placement and quality of MAGs were assessed by MiGA (137) and CheckM (138). The DPANN archaea have undergone such an extensive genome reduction that even the closed complete genomes (e.g., “*Candidatus* Mancarchaeum acidiphilum” Mia14) would have a genome completeness of 82.4% assessed by the above-mentioned method. We thereafter also assessed the relative completeness of each MAG based on the presence of 974 single-copy marker genes of “*Candidatus* Mancarchaeum acidiphilum” Mia14 (80) identified with CheckM (138).

### Pan-genome and comparative genomic analyses.

Available DPANN genomes in the public databases (GenBank, ggKbase, and JGI-IMG) were collected (*n* = 515, excluding genomes with contamination over 5%) for comparative genomic analysis with the 41 novel MAGs. Coding sequences in each genome were predicted using Prodigal v. 2.6 (139). OrthoFinder v1.1.4 (140) was then used to cluster the protein sequences in each genome into orthogroups (with default parameter). A representative sequence from each orthogroup was then used for functional annotation by eggNOG-mapper v. 2.0 (141) (default parameters: E value < 10^−3^, bit score > 60). Spearman rank correlation tests and principal-component analysis (PCA; applying Bray-Curtis distance) were performed in OriginPro 2020b (OriginLab, Northampton, MA, USA). ANOSIM (analysis of similarity) in the vegan R package (142) was used to determine whether there was a significant (*P* < 0.05) difference between the groups and within groups with Bray-Curtis distance. An unpaired (between-group) *t* test was performed with GraphPad Prism v 9.0 (GraphPad, San Diego, CA, USA).

Model extrapolation of the pan-genome and core genome was conducted with the BPGA pipeline v.1.3 (143) applying USEARCH v.11.0 (http://www.drive5.com/usearch/) for clustering gene families with a 30% sequence identity cutoff and 300 random permutations of genomes to prevent bias in the sequential addition of new genomes. The size of the pan-genome was fitted into a power-law regression function, *Ps* = κ*n*γ, with a built-in program of the BPGA pipeline (143) (*Ps* is the total number of gene families; *n* is the number of analyzed genomes; γ is a free parameter). The pan-genome was defined as being “open” (which meant that each added genome would contribute some new genes and the pan-genome would increase) in cases where the calculated exponent γ had an outcome falling in the range between 0 and 1, which was often observed in prokaryotic pan-genomes (144), and the openness of the pan-genome increased as the exponent γ was closer to 1 (tends to be linear). However, if the exponent γ had an outcome smaller than 0, then the pan-genome was defined as being “closed” (which meant that the size of pan-genome is relatively constant as new genomes were added), as observed in Staphylococcus (145).

The size of the core genome was fitted into an exponential decay function, *Fc* = *κc*^−*n*/*τc*^, with a built-in program in the BPGA pipeline (143) (*Fc* is the number of core gene families and *κc* and *τc* are free parameters). OrthoVenn v.2.0 (146) was applied for clustering analysis and creating Venn diagrams based on orthologous clusters. Since no core gene was found in the tested genomes and many draft genomes in this study lack complete small-subunit (SSU) rRNA genes, we applied CVTree3 (39, 40) for alignment-free phylogeny reconstruction based on whole-genome sequences (k-mer = 4). The tree and the presence and absence pattern of genes were visualized using iTOL (147) with the genomes of *Euryarchaeota* as the outgroup.

Average nucleotide identity (ANI) (37) of each MAG relative to public DPANN genomes (*n* = 515) (Table S2) was calculated with the ANI calculator (37). The percentage of conserved proteins (POCP) (38) between our MAGs and their phylogenetically closest genomes in public database was calculated with DIAMOND (148). The phylogenetically closest genome was defined as the public available genome that shared the most recent common ancestor (MRCA) with corresponding MAG in CVTree3 phylogeny. If the MAG formed a monophyletic clade in the phylogenetic tree, then the available public genome sharing the MRCA with the corresponding MAG and in the most basal position was chosen as the reference genomes. Sequence similarity networks (SSN) of gene families of interest were calculated with EFI-EST Tools (149) (with an E value cutoff of 10^−5^ and an identity cutoff of 35%) following the official online tutorial (https://efi.igb.illinois.edu/efi-est/tutorial.php). Sequences sharing more than 90% identity in SSNs were consolidated into the same “metanode.” The SSNs were finally visualized with the “Organic layout” tool in Cytoscape v. 3.7.1 (150).

### Evolutionary analyses and putative lateral transferred gene prediction.

The gain-and-loss pattern of representative high-quality DPANN genomes, including all currently available complete closed genomes (i.e., “*Candidatus* Mancarchaeum acidiphilum” Mia14, “*Candidatus* Forterrea multitransposorum” CG_2015-17, “*Candidatus* Nanopusillus acidilobi” 7A, Nanoarchaeum equitans Kin4-M, and the DPANN group archaeon LC1Nh), and representative high-quality gapped genomes (with an estimated completeness >90%) of “*Candidatus* Altiarchaeota” archaeon SM1-MSI (30), “*Candidatus* Parvarchaeota” archaeon FK_AMD_2010_bin_5 (34), “*Candidatus* Huberarchaea” archaeon CG_4_10_14_0_8 (31), “*Candidatus* Pacearchaeota” archaeon AR19 (19), “*Candidatus* Woesearchaeota” archaeon AR15 (19), “*Candidatus* Woesearchaeota” archaeon AR20 (19), “*Candidatus* Aenigmarchaeota” archaeon AR5 (19), and “*Candidatus* Diapherotrites” archaeon AR10 (19) was inferred by applying the Dollo parsimony algorithms implemented in the COUNT program (151) (with default parameters). A chronogram for these representative high-quality DPANN genomes with branch lengths reflecting divergence times was inferred using the RelTime method (152, 153) on the whole-genome tree conducted in MEGA X (154) as described previously (155), and up to five calibration points provided by Timetree database (156) were included in order to ensure the accuracy of chronogram inference. Data on solar luminosity (157), fluctuations of atmospheric O_2_ (158) and CO_2_ amount (159–162), and asteroid impacts (Earth Impact Database [http://www.impact-structures.com/database-of-earth-impact-structures/]) in the Timetree database (156) were shown synchronously with estimated divergence times. Identification of putative lateral transferred genes in the genomes of the DPANN archaea was performed with the Integrated Microbial Genomes (IMG) system (excluding contamination-unscreened genomes) (36), which identified genes as putative laterally transferred genes by the following rules: the best BLAST hits (best bit scores) or >90% of the best hit of the tested gene was outside the taxonomic lineage of the corresponding genome (i.e., genomes from another phylum, class, etc.) but with lower-scoring hits or no hits within the lineage.

### Primer alignments and environmental distribution analysis.

Alignments of full-length 16S rRNA genes from high-quality DPANN genomes with 25 archaeon-specific or universal 16S rRNA gene primers (87) were performed with ClustalW (https://www.genome.jp/tools-bin/clustalw) to examine putative mismatches. The environmental distribution and abundance of the DPANN members were assessed with full-length 16S rRNA genes from high-quality DPANN genomes as queries against the Sequence Read Archive (SRA) database (including 93,045 of 16S rRNA gene amplicon data sets from 96 different environments), applying the pipeline described by Lagkouvardos et al. (163).

### Data availability.

The 41 metagenome-assembled genomes (MAGs) generated in this study are available in Genome Warehouse (GWH) in National Genomics Data Center (164) under project accession number PRJCA002651. All genome sequences used in this study can be readily accessed in corresponding databases using the accession numbers provided in Table S1 and Table S2.
